# Non-Isothermal Kinetic Analysis of the Crystallization of Metallic Glasses Using the Master Curve Method

**DOI:** 10.3390/ma4122231

**Published:** 2011-12-20

**Authors:** Joan Torrens-Serra, Shankar Venkataraman, Mihai Stoica, Uta Kuehn, Stefan Roth, Jürgen Eckert

**Affiliations:** 1 Departament de Física, Universitat de les Illes Balears, Cra. De Valldemossa km 7.5, Palma de Mallorca 07122, Spain; 2 IFW-Dresden, Institut für Komplexe Materialien, Helmholtzstraβe 20, Dresden D-01069, Germany; E-Mails: shanky22@gmail.com (S.V.); m.stoica@ifw-dresden.de (M.S.); u.kuehn@ifw-dresden.de (U.K.); j.eckert@ifw-dresden.de (J.E.); 3 IFW-Dresden, Institut für Metallische Werkstoffe, Helmholtzstraβe 20, Dresden D-01069, Germany; E-Mail: s.roth@ifw-dresden.de (S.R.); 4 TU-Dresden, Institut für Werkstoffwissenschaft, Helmholtzstr 7, Dresden D-01069, Germany

**Keywords:** metallic glasses, crystallization kinetics, calorimetry

## Abstract

The non-isothermal transformation rate curves of metallic glasses are analyzed with the Master Curve method grounded in the Kolmogorov-Johnson-Mehl-Avrami theory. The method is applied to the study of two different metallic glasses determining the activation energy of the transformation and the experimental kinetic function that is analyzed using Avrami kinetics. The analysis of the crystallization of Cu_47_Ti_33_Zr_11_Ni_8_Si_1_ metallic glassy powders gives E_a_ = 3.8 eV, in good agreement with the calculation by other methods, and a transformation initiated by an accelerating nucleation and diffusion-controlled growth. The other studied alloy is a Nanoperm-type Fe_77_Nb_7_B_15_Cu_1_ metallic glass with a primary crystallization of bcc-Fe. An activation energy of E_a_ = 5.7 eV is obtained from the Master Curve analysis. It is shown that the use of Avrami kinetics is not able to explain the crystallization mechanisms in this alloy giving an Avrami exponent of n = 1.

## 1. Introduction

Metallic glasses are systems with a disordered structure similar to those in liquids. For this reason the classical treatment of the nucleation and growth phenomena and the crystallization kinetic models generally applied to liquid-solid phase transformations may also be valid in this kind of systems. Beyond the existing models, the best description of kinetics of phase transformation is given by the so-called Kolmogorov-Johnson-Mehl-Avrami (KJMA) theory [1,2,3,4,5]. This model allows calculating the evolution of the transformed volume fraction along the transformation. Basically a phase transformation proceeds through two different process nucleation of the new phase in the parent one and growth of these nuclei. Both phenomena are described in the Classical Nucleation Theory [6] by models that depend on fundamental thermodynamic quantities such as Gibbs free energy difference between the two phases, viscosity of the melt and interfacial energy between both phases, all of them temperature dependent [7,8,9]. However, satisfactory results in describing the phase transformation can also be obtained using simpler approximations as a constant activation energy model, where the energy to overcome the potential barrier for nucleation and growth is considered independent of temperature [10,11,12,13]. The crystallization mode in metallic glasses is in general a primary crystallization of a new phase with a composition that differs from the parent phase. However, it has been shown that the existing models for polymorphic transformations can also be used in describing the primary crystallization [14]. The knowledge of the kinetics of crystallization of metallic glasses is a key point in order to design controlled procedures for the improvement of the properties that depend on the microstructure. However, from the basic research point of view, it helps to validate the proposed models for phase transformations.

In this paper we present a method grounded in the KJMA framework to analyze the crystallization kinetics of non-isothermal transformation curves obtained from calorimetric measurements and we applied it to the study of the transformation mechanisms of two different amorphous metallic alloys. The advantages and limitations of this method are discussed in these case studies, which may be seen as paradigms of different types of primary crystallization transformations.

## 2. Theoretical Basis

### 2.1. KJMA Constant Activation Energy Model

In general the analysis of the kinetics of a phase transformation in a material is based on the two following main assumptions that are encountered in innumerable publications:

(1) The transformation rate at time *t* during a reaction, *dx/dt*, can be expressed as a product of two functions, one depending solely on the temperature, *T*, and the other depending solely on the fraction transformed *x*:

(1) $$\frac{dx}{dt}=k(T)f(x)$$

(2) The temperature dependent function, called the rate constant, follows an Arrhenius type dependency.

(2) $$k(T)=k_{0}\text{ exp}\left(\frac{-E_{a}}{k_{B}T}\right)$$

where *k_0_* is a pre-exponential factor, *E_a_* the apparent activation energy and *k_B_* the Boltzmann constant. Thus, to describe the progress of the reaction at all temperatures and for all temperature-time programs, the function *f(x)*, and the constants *k_0_* and *E_a_* need to be determined. In general, the reaction function *f(x)* is unknown at the outset of the analysis. A range of standard functions which represent particular idealized reaction models have been proposed (see, e.g., [15]). The kinetics of transformations that occur through nucleation and growth of particles of a product phase in a parent phase under isothermal annealing conditions is described by the so called simplified isothermal KJMA equation:

(3) $$x=1-\text{exp}\left[-{\left(kt\right)}^{n}\right]$$

where *k* incorporates the rates of nucleation and growth and *n* is the Avrami exponent. This equation, together with Equation (2), describes the volume fraction of the transformed material, *x*, as a function of time, *t*, and annealing temperature, *T*, in terms of *n* and *E_a_*, since the reaction function *f(x)* becomes (by differentiation of Equation (3)):

(4) $$f(x)=n(1-x){\left[-\text{ln}(1-x)\right]}^{\frac{n-1}{n}}$$

The Avrami exponent depends on the nucleation mechanisms and the growth morphology. It can be written as: n = n_I_ + d·n_g_. The nucleation index, *n_I_*, governs the time dependence of the number of nuclei per unit volume of untransformed material, *N*, as a function of time, $N\propto t^{n_{I}}$ . That is, the nucleation contribution is *n_I_* = 0 for pre-existing nuclei (or site saturation) and it is n_I_ = 1 for time independent nucleation rate. The growth index, *n_g_*, is *n_g_* = 1 for interface-controlled growth and *n_g_* = 1/2 for diffusion-controlled growth. The dimensionality of the growth, *d*, is *d* = 1,2 or 3 for one-, two-, or three-dimensional growth, *i.e.*, where the particles of the product phase grow outwardly like needles, disks or spheres, respectively [10]. One can assume that the nucleation rate per unit volume of untransformed material, *I*, a time-independent growth rate, *u* and a temperature dependent diffusion coefficient, *D* have an Arrhenian temperature dependence such as Equation 2 with *E_I_*, *E_u_* and *E_D_* the activation energy for nucleation, growth and diffusion respectively. In both situations, the apparent activation energy is shared by the nucleation contribution and the growth contribution:

(5) $$E_{a}=\frac{n_{I}E_{I}+d\cdot E_{u}}{n},\;\text{if}\;{\text{n}}_{g}=1$$

(6) Ea=nIEI+d2⋅EDn, if ng=1/2

The difficulties in treating non-isothermal reactions are mainly due to the independent variations of growth and nucleation rate with temperature. Therefore, the KJMA equation is in general not a valid description of transformation kinetics occurring under non-isothermal conditions. However, a non-isothermal transformation consisting of nucleation and growth can still be described by the KJMA equation if it meets the following conditions: (a) all nucleation occurs during the early stage in the process leading to the so-called site saturation and, for the rest of the time, only growth is significant; (b) the growth rate depends only on instantaneous temperature and is independent of time; (c) nucleation is random. The first condition also implies that the transformation rate depends only on the state variables *x* and *T* and not on the thermal history. Avrami [1,2] concluded, in his classic studies on kinetics of phase change, that Equation (4) can be generally applied to a non-isothermal transformation if temperatures and concentrations are within the isokinetic range, in which the ratio of growth rate to the probability of formation of growth nuclei per germ nucleus per unit time becomes a constant. The definition of an isokinetic transformation was further modified by Cahn [16]. This leads to the concept of addivity [6]. Assuming that Equation (1) can be extended to a heat treatment with constant heating rate *β*, we may write:

(7) $$\frac{dx}{dT}=\frac{1}{\beta }k(T)f(x)$$

As already mentioned, the kinetic of phase change is mainly described by two parameters: the activation energy and the Avrami exponent. Their determination drives straightforward to the mechanisms controlling the transformation. Different methods have been used in literature in both isothermal and constant heating regimes to obtain these parameters [17,18,19,20].

Let us analyze the relationship between the kinetic functions in both isothermal and constant heating regimes. Redefining the kinetic function from Equation (4) for isothermal conditions as $P_{iso}(x)\equiv \frac{1}{k_{0}f(x)}$ then the Avrami equation is:

(8) $$P_{iso}(x)=\frac{P_{iso,0}{\left[-\text{ln}(1-x)\right]}^{\frac{1-n}{n}}}{n(1-x)}$$

with *P_0,iso_* = *1/k_0_* a characteristic time. By integration is obtained:

(9) $$G_{iso}(x)=\int_{0}^{x}P_{iso}(x)dx=P_{iso,0}{\left[-\text{ln}(1-x)\right]}^{\frac{1}{n}}$$

By analogy, the continuous heating kinetic function is *P(x)* and has the same functional dependence as the isothermal one differing only by a constant factor *C(n,E_I_,E_u_)* that only depends on the Avrami exponent and the activation energies for nucleation and growth [21]. Then, the kinetic functions under both conditions are related by: *P_iso_ (x) = C(n,E_I_,E_u_)P(x)* and *G_iso_ (x) = C(n,E_I_,E_u_)G(x)*.

Until now we have only considered the case where the nucleation frequency and growth rate were constant through all the transformations. Often, the Avrami exponent depends on the transformed fraction, *n(x)*, indicating that nucleation frequency and/or growth rate increases or decreases during the transformation or that it is not a unique mechanism which is controlling the reaction. A change in the growth mechanism may occur, from an interface-controlled growth to diffusion-controlled growth. Also, the change on matrix composition leads to a decrease in the Avrami exponent such as found in the soft impingement effect [14]. For that reason, the determination of the experimental kinetic function is preferable rather than only the Avrami exponent.

### 2.2. Master Curve Method

The Master Curve method (MCM) [22,23,24] has been developed to study the crystallization under non-isothermal regime. This method is based on the isokinetic hypothesis. As the function *G(x)* is independent from the thermal path:

(10) $$G(x)=\int_{0}^{x}P(x^{\prime })dx^{\prime }=\int_{T_{0}}^{T}\frac{1}{\beta }\text{exp}\left(\frac{-E_{a}}{k_{B}T^{\prime }}\right)dT^{\prime }$$

for any pair of heating rates *β_i_* and *β_j_* we can write:

(11) $$\int_{T_{0}}^{T_{i}}\frac{1}{\beta }\text{exp}\left(\frac{-E_{a}}{k_{B}T^{\prime }}\right)dT^{\prime }=\int_{T_{0}}^{T_{j}}\frac{1}{\beta }\text{exp}\left(\frac{-E_{a}}{k_{B}T^{\prime }}\right)dT^{\prime }$$

That is, a calorimetric curve can be transformed from a heating rate *β_i_* to a heating rate *β_j_* finding for every original temperature *T_i_* a transformed temperature *T_j_* which ensures the equality. Moreover, from Equation (10) the transformed fraction at certain original temperature at the original heating rate is the same to the transformed fraction at the transformed temperature for the new heating rate *x(T_i_,β_i_) = x(T_j_,β_j_)*. Equation (11) can be applied to a set of *p* DSC curves at different heating rates $\left[\frac{dx}{dT}{|}_{i},\beta_{i},T_{i}\right]$ with *i* = 1, 2, …, *p* to transform them to *p* curves with the same equivalent heating rate *β_eq_*, $\left[\frac{dx}{dT}{|}_{eq},\beta_{eq},T_{eq}\right]$ for each value of the activation energy. Following an iterative procedure for a range of values of the activation energy described in Reference [23], the differences between them are minimized and the optimal value of *E_a_* which leads to the best overlap of the *p* transformed curves can be found. Then, the master curve is an average curve of all the converted curves, $\left[\frac{dx}{dT}{|}_{eq},\beta_{eq},T_{eq}\right]$ , that contains the kinetic information. The main advantage of this method over the Kissinger's one is that works over all the points of the curve while the other only takes one point into account (the maximum of the curve) and thus, reduces the uncertainty. Introducing the master curve and the activation energy in Equation (10) the experimental constant-heating-rate kinetic function is obtained:

(12) $$P(x)=\frac{1}{\beta_{eq}}{\left[\frac{dx}{dT}\right]}_{eq}^{-1}\text{exp}\left(\frac{-E_{a}}{k_{B}T}\right)$$

which can be compared to those obtained by the Avrami kinetic model and calculate the Avrami exponent. In the case of non-constant Avrami exponent transformations, *n* is determined locally by fitting the experimental kinetic function by the non-isothermal analogous of Equation (8) in a certain range of *x*.

### 2.3. Transformation Rate Curves and Calorimetric Experiments

The calorimetric signal is a mixture of two contributions: the power released by the alloy when transforming from the disordered state to a partially crystalline state, and the difference in the heat capacity between the amorphous parent alloy and the two phases (crystalline and residual disordered matrix). These two contributions to the total calorimetric signal must be separated before performing any calorimetric study [25]. It has been shown that the overall exothermic DSC signal, *dQ/dt*, may be shared between the instantaneous crystallization enthalpy change, *dΔH/dt*, that is proportional to the transformation rate, *dx/dt* (note that here *x* is the primary transformed fraction), and the heat capacity contribution, *dQ_ΔCp_/dt*, which is proportional to the transformed volume fraction *x* (considering that the overall specific heat of the specimen is given by the linear interpolation of the specific heats of the undercooled melt and the nanocrystalline phase) [26]. The relationship between these quantities is given by:

(13) $$\frac{dQ}{dt}=\frac{d\Delta H}{dt}+\frac{\Delta C_{p}dT}{dt}x=\Delta H\frac{dx}{dt}-\beta \Delta C_{p}x$$

The two contributions to the signal were separated using an iterative procedure [27,28].

## 3. Case Studies

### 3.1. Cu-Based Metallic Glasses

**Figure 1:**
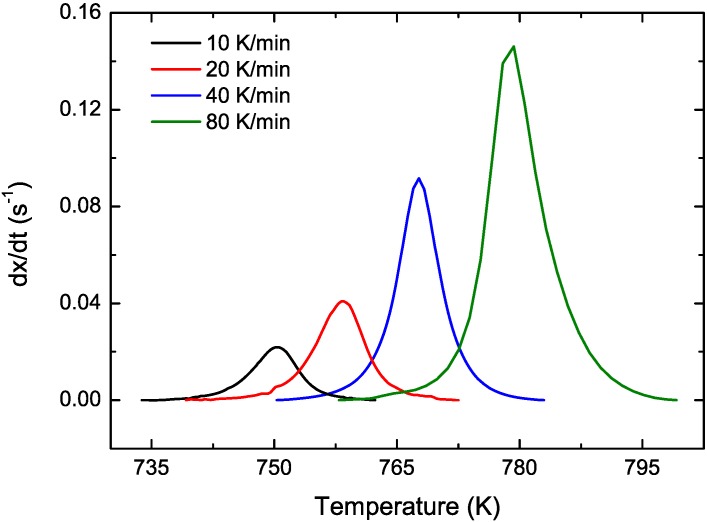
Experimental transformation curves for different heating rates for Cu_47_Ti_33_Zr_11_Ni_8_Si_1_ alloy.

**Figure 2:**
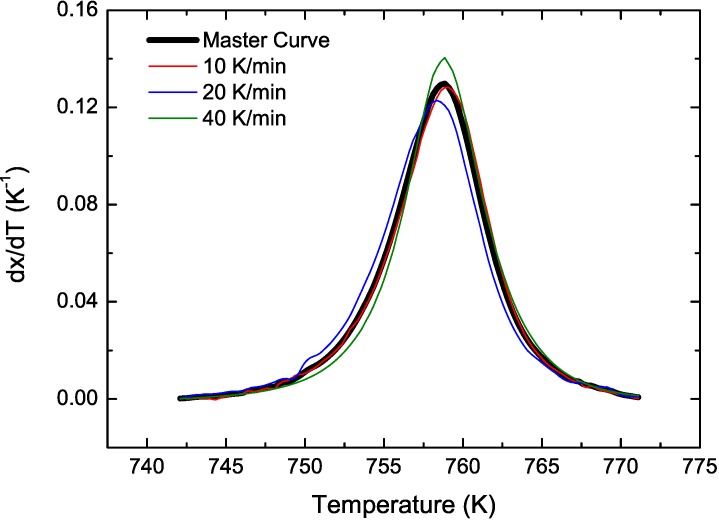
Transformed experimental curves (in color) and Master Curve (black line) for Cu_47_Ti_33_Zr_11_Ni_8_Si_1_ alloy.

**Figure 3:**
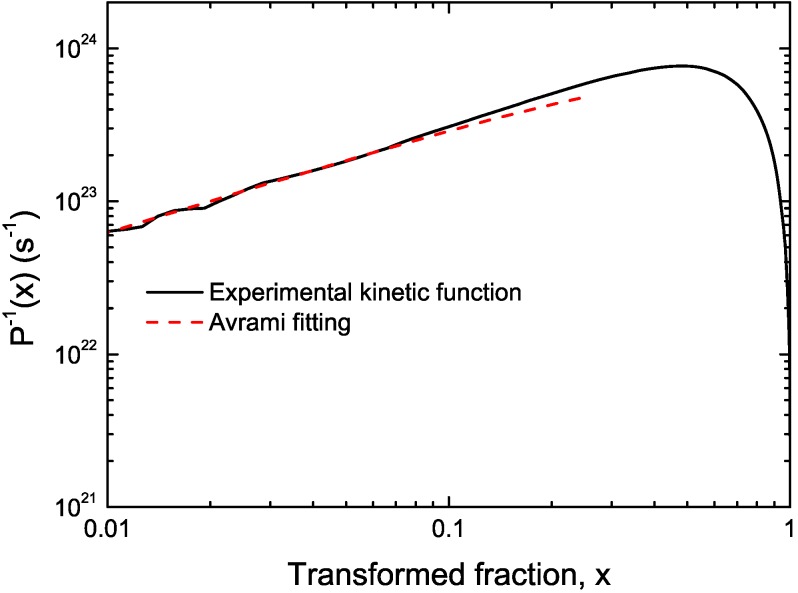
Experimental kinetic function (solid black line) and fitting Avrami kinetic function (dashed line) for Cu_47_Ti_33_Zr_11_Ni_8_Si_1_ alloy.

**Figure 4:**
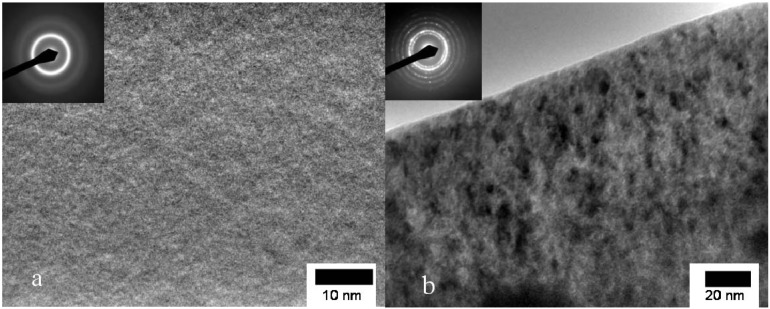
TEM micrographs of Cu_47_Ti_33_Zr_11_Ni_8_Si_1_ alloy (**a**) as-quenched powder and (**b**) after heating up to 785 K.

CuTiZr bulk metallic glasses have been widely studied due to their high glass forming ability and their potential use as structural materials [29,30,31]. In our study amorphous powders of Cu_47_Ti_33_Zr_11_Ni_8_Si_1_ were obtained by Ar gas atomization. Microstructural characterization was carried out on ion-milled powder samples using a JEOL 2000 FX TEM operated at 200 kV accelerating voltage. The DSC curves have been obtained using a DSC7 PE calorimeter using Al pans and Ar as flowing gas. The curves at 40 K/min show a single primary crystallization peak at 762 K preceded by a supercooled liquid region of about 62 K. The XRD studies in this alloy show that the first crystallization event corresponds to the precipitation of the intermetallic Cu_51_Zr_14_ phase [32]. DSC curves at different heating rates from 10–80 K/min have been recorded. In Figure 1 the *dx/dt* curves after removing the heat capacity contribution at different heating rates are presented. The curves are transformed to the equivalent heating rate of 20 K/min as shown in Figure 2. The best overlap was obtained for an activation energy of *E_a_* = 3.8 eV comparable with the values obtained using other methods *E_a_* = 3.5 eV with Kissinger and *E_a_* = 3.7 eV with isothermal methods [32]. The corresponding kinetic experimental function is plotted in Figure 3. The fitting with the Avrami kinetic function in the range of 2% < *x* < 8% leads to a value of the Avrami exponent of *n* = 3.2 and *P_0_* = 4.68 × 10^−23^ s. Different interpretations could arise from this value. A first possibility would suggest a 3D interface-controlled growth of quenched-in nuclei. However, this option may be refuted by the TEM microstructural investigations. As observed in the presented TEM pictures (Figure 4), no previous nuclei are found in the as-quenched samples proving that nucleation takes place during the transformation [32,33]. A more reasonable interpretation suggests a transformation controlled by a nucleation and diffusion-controlled growth mechanism. In this case the *n_I_* > 1 which correspond to an increasing nucleation frequency. The kinetic analysis extracted from Master Curve method is compatible with the interpretations of the transformation mechanisms found using the differential Avrami method from isothermal measurements reported in previous works [32].

### 3.2. Cu Containing Fe-Based Alloy

**Figure 5:**
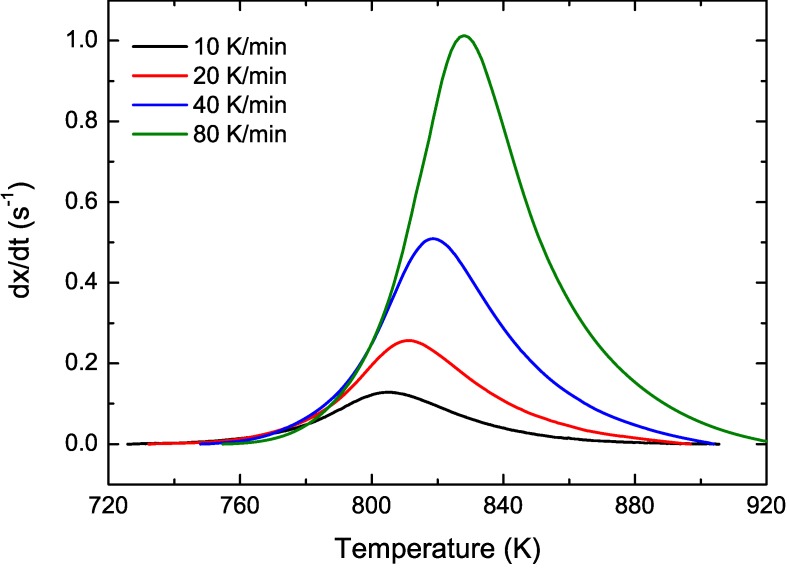
Experimental transformation curves for different heating rates for Fe_77_Nb_7_B_15_Cu_1_ alloy.

**Figure 6:**
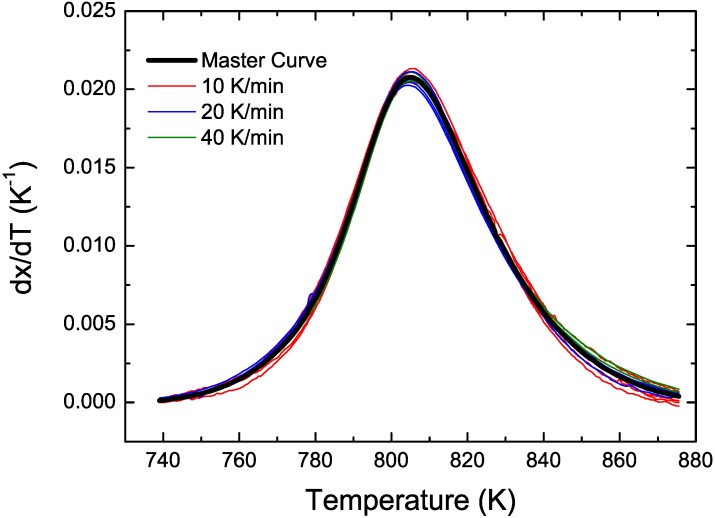
Transformed experimental curves (in color) and Master Curve (black line) for Fe_77_Nb_7_B_15_Cu_1_ alloy.

**Figure 7:**
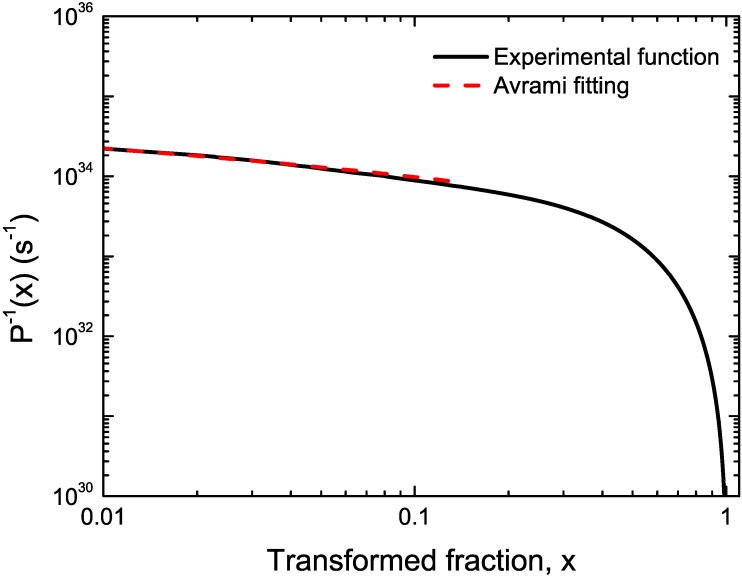
Experimental kinetic function (solid black line) and fitting Avrami kinetic function (dashed red line) for Fe_77_Nb_7_B_15_Cu_1_ alloy.

**Figure 8:**
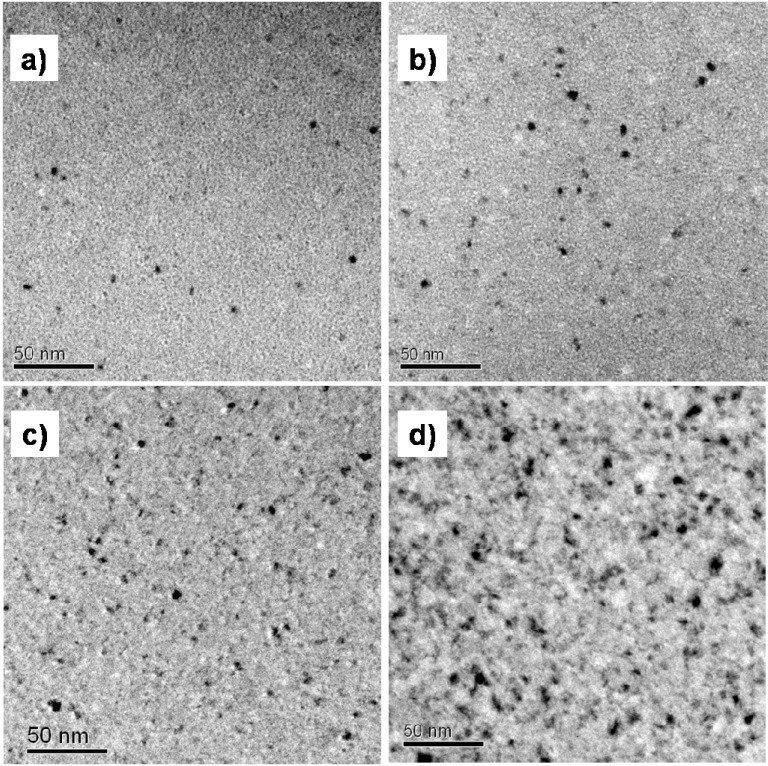
TEM micrographs of Fe_77_Nb_7_B_15_Cu_1_ alloy after heating to different temperatures at 5 K/min up to (**a**) 765 K; (**b**) 788 K; (**c**) 818 K; and (**d**) 873 K.

It is well known that minor additions of Cu to the Nanoperm type alloys favor the nanocrystallization of bcc-Fe through the heterogeneous nucleation promoted by the Cu clustering. In this case ribbons of Fe_77_Nb_7_B_15_Cu_1_ were prepared by melt-spinning technique at 47 m/s in fully amorphous state [34]. The microstructural observations were carried out in a Philips CM30 microscope. The DSC curves have been obtained using a DSC7 PE calorimeter using Au pans and Ar as fluxing gas. In this alloy the calorimetric studies do not show a trace of glass transition previous to the primary crystallization. The calorimetric peak is very broad and extended over a wide temperature range as can be observed in Figure 5. The experimental calorimetric curves recorded at different heating rates are transformed using the Master Curve method to an equivalent heating rate of 10 K/min. The value of activation energy that leads an optimum overlap of the transformed curves (Figure 6) is *E_a_* = 5.7 eV. This value is typical for this kind of alloys and has been also found in other Fe(Co)NbBCu alloys [34]. The experimental kinetic function is plotted in Figure 7 together with the Avrami kinetic function fitting in the first stages (x < 10%). In this case we obtain an Avrami exponent *n* = 0.8 and *P_0_* = 1.42 × 10^−34^ s. This abnormal low value of *n* at the beginning of the transformation is difficult to interpret by the KJMA model. In order to explain the transformation mechanisms microstructural information is required. The TEM micrographs obtained after heating up to different crystallization stages (Figure 8) indicate that nucleation is present over the entire process showing a slight decrease with temperature [35] and nanocrystals with a final size of about 4–6 nm. In this case, the determination of the Avrami exponent with the fitting of constant *n* Avrami kinetic function is not able to explain the observed microstructure. A simple mathematical model has been proposed to explain the observed microstructure [35]. Other authors have used other approximations as an instantaneous growth, together with a decreasing nucleation to explain the low *n* values encountered in these alloys [36]. In comparison to these types of alloys, which have low glass forming ability and are only quenched in ribbon form, high glass forming ability Fe-based alloys, which have been obtained in as BMGs with diameters up to 6 mm, have been reported in the last years [37]. It is worth noting that the primary crystallization product is no longer bcc-Fe but a fcc-Fe_23_B_6_ structure with large unit cell which it is believed that this is the main reason for the high GFA [38,39,40]. However, only alloys with high content of metalloid atoms (more than 20%) show this behavior [41]. In a previous study on the crystallization kinetics of a Fe_65_Nb_10_B_25_ metallic glass [42], the mechanisms controlling the transformation were determined to be the nucleation and interface-controlled growth changing to a diffusion-controlled growth as the transformation advances and *I* and *u* become complex functions of *T* and *x* [14]. These findings differ from the results in our FeNbBCu alloy proving a strong relation between the nanocrystallization kinetics and the primary crystallization products.

## 4. Conclusions

The Master Curve method has shown to be a useful tool to analyze the crystallization kinetics of metallic glasses. This method is not only limited to find the activation energy value as is the case for other non-isothermal methods, but also gives the experimental kinetic function, which can be further used to determine the crystallization mechanisms by fitting to the Avrami kinetic function combined with the help of microstructural observations. The main advantages of this method over the existing ones are the use of the whole calorimetric curve instead of only one specific point (peak, onset), and the possibility of the determination of the overall kinetic function instead of a particular Avrami exponent that is in general not constant over the entire transformation.

The method has been applied to two different case studies showing different crystallization mechanisms. The transformation of a Cu-based metallic glass is driven by an accelerating nucleation process and a diffusion-controlled growth. Finally, the Fe-Nb-B-Cu alloy shows a transformation behavior that cannot be described within the KJMA model.
